# Infant’s thermal balance and the evolution of the human breast – a proof-of-concept study

**DOI:** 10.1017/ehs.2025.10024

**Published:** 2025-12-11

**Authors:** Tiina Kuvaja, Tiina Väre, Sirkka Rissanen, Hannu Rintamäki, Petri Lehenkari, Juho-Antti Junno

**Affiliations:** 1 Archaelogy, Faculty of Humanities, University of Oulu, Oulu, Finland; 2 Finnish Institute of Occupational Health, Oulu, Finland; 3 Department of Anatomy, Medical Research Center Oulu, University of Oulu and Oulu University Hospital, Oulu, Finland

**Keywords:** mammary tissue, breast evolution, thermoregulation, lactation

## Abstract

The distinct size and shape of breasts in women is a uniquely human trait. This trait has no conclusive explanation as it is not a requirement for milk production. Additionally, breasts are already enlarged at puberty, which is usually long before the first pregnancy. We hypothesized that the perennially enlarged human breasts were potentially developed to support infant’s thermal balance by providing increased warming surface in skin-to-skin contact.

To test the hypothesis, we measured breast surface temperature to explore their heating capacity and resilience to temperature changes in an environmental conditions laboratory. Volunteers, divided in groups of nursing women, non-nursing women, and men, were exposed to three temperatures: 32°C, 27°C, and 18°C. The exposure time in each temperature was 20 min. The changes in breast surface temperature were recorded by thermal imaging camera. Data were analysed using Kruskal–Wallis tests. Breastfeeding women had overall higher mammary surface temperature compared to other groups. Furthermore, nursing women had distinct resilience against cooling environment: they lost the average of 2.5°C of their mammary surface temperature, whereas other study groups lost 4.3°C and 4.7°C of surface temperature, respectively. This proof-of -concept study clearly indicated the potential of the nursing women’s breasts to support infant’s thermal balance.

## Graphical abstract

**Figure:**
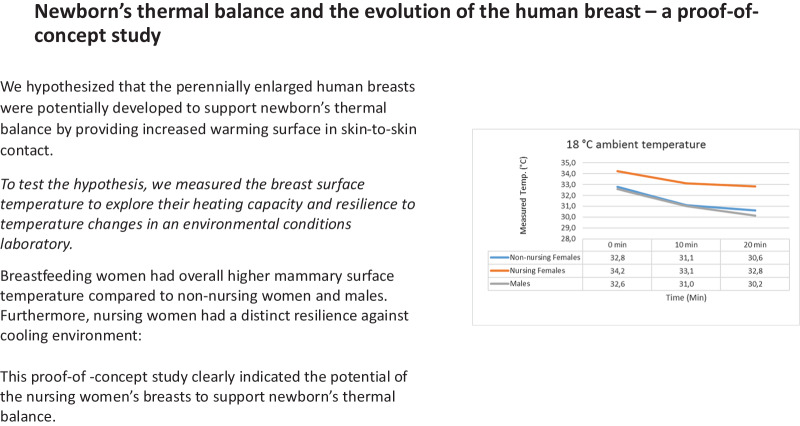

## Social media summary

*We suggest that human breasts were potentially developed to support infant’s thermal balance.*

## 1. Introduction

Although there is considerable variation in the breast tissue volume (J. Anderson et al., 2013), the prominent shape and size of female breasts is a special trait from an evolutionary perspective. It seemingly does not serve any anatomical or physiological function (Scutt et al., 1997), but instead has clear drawbacks as increased breast size is correlated with several health issues such as back pain (Benditte-Klepetko et al., 2007). Unlike most other mammals and especially other primates (Cant, 1981; Short, 1979), human females retain quite prominent size and shape of mammary tissues starting from puberty (Russo & Russo, 2004). It is suggested that permanent breasts are a side effect of subcutaneous fat tissue increase ‘during the *Homo erectus* stage of human evolution’ and human breasts have developed due to the deposition of periglandular adipose tissue at puberty (Pawłowski, 1999).

In this study we wanted to investigate if perennially enlarged breasts could have been developed to support the thermal balance of the infant’s body, thereby compensating for the loss of insulating fur and premature labour in our species (Byaruhanga et al., 2005; Galligan, 2006; Pinheiro et al., 2014). Lactating females of other species such as vervet monkeys have higher body temperatures than non-lactating ones (McFarland et al., 2024), a phenomenon that may be related to thermoregulation of newborns (Lezama-García et al., 2022). It is suggested that the loss of fur appeared approximately 2 Mya together with the arrival of *Homo* and warming climate after the cooling period at 2.5 Mya (Dávid-Barrett & Dunbar, 2016; Demenocal, 1995). This would provide a straightforward evolutionary mechanism as an explanation for unique morphology of women’s mammae: their tissues act as a thermoregulatory heat source for the infant, increasing shielding surface in skin-to-skin contact, thus preventing terminal heat loss, and adding body heat surface area amplifying warming effect. Compared to chimpanzees, human infants require 8.7% more energy during the first 18 months of their life (Foley & Lee, 1991) and it is suggested that this is mainly due to encephalization and thermoregulation of the head (Pawłowski, 2005). The role of increased adipose tissue is recognized as an important life-history strategy of human infants during early evolution as it provided energy storage and a mechanism for thermoregulation (Kuzawa, 1998). Therefore it would not be surprising that deposition of adipose tissue in the mother could also have an important role for the infant’s survival.

In the current understanding, perennially enlarged breasts seemingly do not serve any obvious function. The reason for the unique size and shape is the accumulation of subcutaneous fat tissue, not the glandular lobules (Vandeweyer & Hertens, 2002). In contrast, the other great apes have prominent breasts only during the initial phase of lactation, and this is caused by increased activity on glandular tissue, that is, lactation that potentially also increases the breast temperature. Human females, on the other hand, have prominent mammae all through adulthood. No comprehensive explanation has been found to this morphological adaptation. Common hypotheses speculate that enlarged breasts act as a sexual signal indicating fertility and fitness (P. Anderson et al., 1983; Caro, 1987; Jasieńska et al., 2004). However, in many cultures breasts are not sexualized specifically (Dettwyler, 1995; Ford & Beach, 1952). In the relatively recent review study by Pawłowski and Żelaźniewicz (2021) it was proposed that breasts originally appeared as a by-product of other evolutionary processes such as an increase in subcutaneous fat tissue and possibly developed further by sexual selection as enlarged breasts may have been a major indication of fitness.

In this proof-of-concept study we wanted to investigate if the female breast tissue would have a distinct response to changing temperature. This could indicate a potential stabilizing thermal effect for the infant and especially the newborn, besides just the obvious increased surface area of warmth and insulation of women’s breasts. There are some mentions in the literature of mammary tissues having changed temperature according to newborns’ thermal needs during skin-to-skin -contact (Chen et al., 2000; Karlsson et al., 1995; Kimura & Matsuoka, 2007). It is also noted that lactation influences breast skin temperature (e.g. Gouveia et al., 2019) and it is even noted that relatively large changes in mammary metabolic rate and mammary temperature take place in early pregnancy (Burd et al., 1977).

## 2. Materials

Twenty-seven volunteers formed three subgroups: non-breastfeeding women (*N* = 12), currently breastfeeding women (*N* = 8), and men (*N* = 7). Participants of this study were 20–40 years of age. This age category was selected to represent typical age of nursing women and to avoid individuals undergoing pubertal or menopausal periods. The group of non-breastfeeding women included both women who had not given birth and those who had stopped breastfeeding at least one year prior to our study. Our test setting produced 243 data points in three different temperature conditions, 18°C, 27°C, and 32°C.

The study protocol follows the Declaration of Helsinki and has been approved by the Ethics Committee of the Northern Ostrobothnia Hospital District. Consent to attend to this study was obtained from volunteers and formal agreements for tests were made with volunteer signatures.

## 3. Methods

Our study was designed and carried out in the climatic chamber of the Finnish Institute of Occupational Health. Method testing and development started 3 July 2017 and the study was completed by December 2022. Temperature environments were created with the laboratory’s climatic chamber. In the climatic chamber, air temperature, velocity, and humidity (30%) were accurately controlled. Skin temperatures of the test subjects were measured by laboratory’s thermal imaging camera (FLIR SYSTEMS ThermaCAM PM695 PAL) and processed with FLIR ThermaCAM Researcher™ program.

Volunteers were exposed to three temperatures in the climatic chamber: 32°C, 27°C, and 18°C. The test temperatures were chosen to represent normal human environment temperatures, subject to practical limitations. Although 27°C is a thermoneutral temperature for human, representing the mean day temperature of the African savannah, the night temperatures may fall well below the 18°C and daytime temperatures can rise above 32°C. Considering the well-being of the volunteers – especially the nursing mothers – the extreme temperatures were chosen to be represented by 18°C and 32°C.

Thermal imaging was performed with volunteers standing in a standard anatomical position dressed in lightweight garments covering only the lower body. The exposure time at each temperature was 20 min. The temperature changes in breast surface were recorded using thermal imaging at 0 and 10-min points and at the end of the exposure, that is, the 20-min point. Between different test settings, the volunteers spent 30 min at a typical room temperature of 21–23°C. The captured thermal images were analysed with FLIR ThermaCAM Resercher™ program using a targeted area emission tool, which measures overall temperature emission of the pointed area, which was chosen to be the right breast area.

The groups were compared in terms of the temperature drop between the measurement points utilizing standard statistical tools (IBM SPSS Statistics 27). The surface temperature measure after 20 min at 32°C was used as a reference point as it generally offered the highest surface temperature value. While the variances of the groups are homogeneous, the data failed Shapiro–Wilk and Kolmogorov–Smirnov tests and the sample size is small, which is why Kruskal–Wallis tests (95% confidence level) were used.

## 4. Results

The change in mean surface temperatures is presented in Table 1. The comparisons across the groups at the end of the experiment (20 min at 18°C) indicate the similarity of temperatures between men and non-nursing women (Kruskal–Wallis pairwise comparison adjusted by Bonferroni correction *p* = 1.000). Both men (*p* = 0.000) and non-nursing women (*p* = 0.003) are different from the nursing women.

**Table 1.** The mean surface temperatures in groups of non-nursing and nursing women and men after exposure to each temperature setting. The groups (1–3) are similar (*p* = 0.916) in terms of ages

Group	*N*	Mean age (years)	SD (age)	Mean 20 min in 32°C	Mean 20 min in 27°C	Mean difference between 32°C and 27°C	*p* value	Mean 20 min in 18°C	Mean difference between 32°C and 18°C	*p* value
Non-nursing women	12	31.0	9.3	35.1 ± 0.6°C	33.5 ± 0.6°C	−1.6 ± 0.5°C	0.003	30.4 ± 0.9°C	−4.7 ± 0.6°C	0.001
Nursing women	8	29.9	2.6	35.4 ± 0.6°C	35.3 ± 1.7°C	−0.1 ± 1.7°C		32.9 ± 0.8°C	−2.5 ± 0.7°C	
Non-nursing women	12	31.0	9.3	35.1 ± 0.6°C	33.5 ± 0.6°C	−1.6 ± 0.5°C	1.000	30.4 ± 0.9°C	−4.7 ± 0.6°C	1.000
Men	7	30.9	12.4	34.2 ± 1.1°C	32.7 ± 0.8°C	−1.5 ± 0.6°C		29.9 ± 0.7°C	−4.3 ± 0.9°C	
Nursing women	8	29.9	2.6	35.4 ± 0.6°C	35.3 ± 1.7°C	−0.1 ± 1.7°C	0.020	32.9 ± 0.8°C	−2.5 ± 0.7°C	0.010
Men	7	30.9	12.4	34.2 ± 1.1°C	32.7 ± 0.8°C	−1.5 ± 0.6°C		29.9 ± 0.7°C	−4.3 ± 0.9°C	
All	27	30.6	8.6	34.9 ± 0.9°C	33.8 ± 1.5°C	−1.2 ± 1.2°C		31.0 ± 1.5°C	−3.9 ± 1.2°C	

The decrease in the mean surface temperature after 20 min at 27°C (Table 1) was similar for the groups of non-nursing women and men (*p* = 1.000), but both these groups were statistically significantly different from the group of nursing women (*p* = 0.003 and 0.020, respectively). Finally, after 20 min at 18°C the mean decrease in temperature of the nursing women is significantly different from that of non-nursing women (*p* = 0.000) and men (0.010). The latter two groups are still very similar (*p* = 1.000).

## 5. Discussion

Two significant observations stand out from the data. First, non-nursing women and men had very similar patterns in surface temperature loss (Table 1). Second, nursing women had a distinct resilience against a cooling environment compared to other two groups: during the test the group of nursing women lost an average of 2.5°C from their mammary tissue’s temperature, whereas the other two groups lost averages of 4.3°C and 4.7°C.

These two observations indicate that differences in overall tissue temperature or the resilience against cooling is at least not solely due to the insular abilities of excess adipose tissue per se, but rather it is potentially the product of the activity of the female mammary gland and thoracic vasculature system associated with pregnancy, labour, and nursing (Alex et al., 2020). Men had slightly more resilient heat retention compared to the non-nursing women probably due to greater muscle mass providing a heat source (Guyton & Hall, 2006).

Our findings are in accordance with previous research on mother–newborn skin-to-skin contact; the mother’s skin temperature in skin-to-skin contact is an important factor keeping the infant at stable body temperature (Bystrova et al., 2007). Previous studies have also addressed the potential role of thermoregulation for enlarged breasts (Einon, 2007). Newborns’ demand for an external source of warmth cannot be understated; a robust body of data indicates that even in modern hospital settings newborn hypothermia is a serious risk (Byaruhanga et al., 2005; Galligan, 2006; Pinheiro et al., 2014). Clinical settings have also demonstrated that mammary temperature adjusts according to the infant’s thermal needs (Ludington-Hoe et al., 2006). In previous studies it is noticed that breast skin temperature patterns of lactating women differ from non-lactating ones (Gouveia et al., 2019). Shared body heat has been the most convenient and reliable heat source since before the invention of fire or protective clothing. The morphology of the female breast provides a large surface for skin-to-skin contact as the prominent shape and elasticity of the breast multiplies the contact area compared to a planar surface.

This proof-of-concept study clearly demonstrated distinct heat retention and radiation capabilities of mammary tissues that could potentially protect an infant from temperature fluctuations in the absence of insulating body hair or fully developed thermogenesis. A recent study on wild primates by McFarland et al. (2024) demonstrated that the body temperatures of lactating females and non-lactating ones have clear differences, potentially associated with the infant’s thermoregulation. Our findings suggest that a newborn’s thermal balance may be connected with the evolutionary origins of perennially enlarged breasts.

The finding is in line with earlier studies suggesting that breast morphology may have evolved as a by-product of coincident evolutionary processes that included an increase in subcutaneous fat tissue and developed further by sexual selection (Pawłowski & Żelaźniewicz, 2021). However, breasts are already enlarged in puberty, usually long before the first pregnancy. It is possible that adipose tissue in mammae has a secondary function as an energy store. Our findings indicate that the adipose tissue is important for increased breast volume and thus the skin-to-skin contact area in general, but the resilience against cooling is produced by the activity of the mammary gland. Apart from thermoregulation, other potential explanations for our findings such as effective milk removal (Gardner et al. 2019) couldn’t possibly provide strong enough selection pressure. Further studies with larger sample sizes and other exposure temperatures are recommended to better understand the observed phenomenon and the evolution of perennially enlarged breasts. In addition, similar studies on other primates, such as breastfeeding and non-breastfeeding female chimpanzees, could shed light on the role of breastfeeding in chest temperatures in general and, consequently, on the origin of perennially enlarged breasts.

## Data Availability

All data are available upon a reasonable request from the corresponding author.

## Research transparency and reproducibility

The authors affirm that this manuscript is an honest, accurate, and transparent account of the study.
